# HJ16 induced resistant virus displays rare natural mutation highlighting the conserved nature of this new CD4bs epitope

**DOI:** 10.1186/1742-4690-8-S2-P33

**Published:** 2011-10-03

**Authors:** Sunita S Balla-Jhagihoorsingh, Betty Willems, Katleen Vereecken, Davide Corti, Leo Heyndríckx, Guido Vanham, The BMGF CAVD UCL Vaccine Discovery Consortium

**Affiliations:** 1 Institute of Tropical Medicine (ITM), Antwerp, Belgium; 2 Institute for Research in Biomedicine (IRB), Bellinzona, Switzerland

## Background

Immunogen development for HIV-1 vaccines can be based on epitope identification of naturally occurring neutralizing antibodies in HIV-1 infected patients. A neutralizing monoclonal antibody, HJ16, was obtained at IRB from a patient recruited at ITM which recognized a new epitope in the CD4bs and neutralized mostly tier 2 strains that were not neutralized by b12[1]. This new CD4bs epitope is not fully characterized yet but could be important for immunogen design. We therefore used a neutralization sensitive virus and after resistance induction compared the resistant and sensitive strains to map important regions for the HJ16 neutralizing activity.

## Materials and methods

HJ16 resistance was induced by culturing the sensitive replication competent VI1090 (CRFO2_AG) strain (IC50 0.10 µg/ ml) in increasing amounts of HJ16 on freshly isolated PBMC until a resistant strain was obtained that was able to replicate in almost 200 µg/ml HJ16. Neutralizing activity was measured using TZMbl and PBMC neutralization assays. Site-directed mutagenesis was carried out to induce the observed mutations into the VI1090 expressing vector.

## Results

Sequencing of the HJ16 sensitive versus the resistant strain revealed a distinct point mutation where the neutral asparagine was replaced by the negative charged aspartic acid. This N276D point mutation is only seen in 0.69% of the group M strains described in the Los Alamos database (total of 1885 viruses, 6 cases with N276D and 7 cases with a polar N276S mutation). Introduction of this mutation into the HJ16 neutralization sensitive construct rendered the mutated strain resistant to neutralization by at least 100 fold higher HJ16 concentration.

## Conclusions

Results show that the mutation in the N-linked glycosylation site at N276 has a distinct influence on sensitivity to the HJ16 CD4bs antibody. Although it is disappointing that a single mutation seems to induce resistance it is obvious that this mutation is quite unique and occurs rarely in natural infection rendering this epitope very suitable for further vaccine development.
